# A DSM-5-based tool to monitor concurrent mood and premenstrual symptoms: the McMaster Premenstrual and Mood Symptom Scale (MAC-PMSS)

**DOI:** 10.1186/s12905-022-01678-1

**Published:** 2022-03-30

**Authors:** Benicio N. Frey, Olivia R. Allega, Maha Eltayebani, Sabrina K. Syan, Jeronimo Mendes-Ribeiro, Luciano Minuzzi

**Affiliations:** 1 Women’s Health Concerns Clinic, St. Joseph’s Healthcare Hamilton, 100 West 5th Street, Suite C124, Hamilton, ON L8N 3K7 Canada; 2 Mood Disorders Program, St. Joseph’s Healthcare Hamilton, Hamilton, ON Canada; 3 Department of Psychiatry and Behavioural Neurosciences, McMaster University, Hamilton, ON Canada; 4 Department of Neuropsychiatry, Faculty of Medicine, Alexandria University, Alexandria, Egypt

**Keywords:** Premenstrual symptoms, Premenstrual dysphoric disorder, Depression, Major depressive disorder, Bipolar disorder, Diagnosis, Monitoring

## Abstract

**Background:**

Despite high co-morbidity between premenstrual dysphoric disorder and mood disorders, there is a gap of research-based tools to monitor concurrent premenstrual and mood symptoms. In this study, we developed a new DSM-5-based questionnaire to prospectively monitor concurrent premenstrual and mood symptoms.

**Methods:**

Fifty-two females with bipolar or major depressive disorder, ages 16–45, were enrolled in the study. Participants completed two months of prospective symptom charting including the McMaster Premenstrual and Mood Symptom Scale (MAC-PMSS) and the Daily Record of Severity of Problems (DRSP). At the end of the prospective charting, participants also completed the Montgomery-Åsberg Depression Rating Scale (MADRS), Hamilton Depression Rating Scale (HDRS) and the Young Mania Rating Scale (YMRS). The MAC-PMSS was correlated with the DRSP, MADRS, HDRS and YMRS.

**Results:**

All individual items of the MAC-PMSS correlated strongly with the individual DRSP scores (all p < 0.001). The mood section of the MAC-PMSS also significantly correlated with MADRS (r = 0.572; p < 0.01), HDRS (r = 0.555; p < 0.01) and YMRS scores (r = 0.456; p < 0.01).

**Conclusions:**

The MAC-PMSS is a reliable to tool to measure concurrent mood and premenstrual symptoms in women with mood disorders.

## Background

It has been long recognized that women may experience significant emotional or physical symptoms in the late luteal phase of the menstrual cycle (i.e., the week before menses). It is estimated that 30–40% of women experience significant premenstrual symptoms that was classified by the American College of Obstetricians and Gynecologists as “premenstrual syndrome” (PMS) [1, 2]. However, it is estimated that 5–8% of women meet criteria for a severe form of premenstrual syndrome defined as “premenstrual dysphoric disorder” (PMDD), which was included in the 5th edition of the Diagnostic and Statistical Manual of Mental Disorders [3, 4]. Previous studies have found that women with bipolar disorder (BD) and major depressive disorder (MDD) are more likely to experience premenstrual worsening of mood [5, 6]. Mood and anxiety disorders have been shown to be 66% more common in women with PMDD [7]. Choi et al. [8] established that of bipolar type-I females, 23.3% experienced moderate to severe premenstrual symptoms, while 6.7% suffered from PMDD. Of bipolar type-II females, 51.6% experienced moderate to severe premenstrual symptoms, while 22.6% suffered from PMDD. In Blehar et al. [9] study, two-thirds of women with bipolar-I disorder reported premenstrual worsening of mood symptoms.

Large community-based studies have also suggested a link between PMDD and mood disorders. Wittchen et al. [10] in their large survey of 1488 young women, who were followed-up over a period of 4 years, found a 22 ± 9% (12-month and lifetime) co-morbidity rate between mood disorder and PMDD. A study of 3518 women completed by Forrester-Knauss et al. [11] revealed a 24.6% prevalence of major depression in females who screened positive for PMDD and 11.3% in those who screened positive for moderate premenstrual symptoms. We have recently found that females with co-morbid PMDD and BD have higher rates of relapse, rapid cycling, and an earlier onset of bipolar onset closer to menarche [12]. We have also recently found that women with co-morbid PMDD and BD appear to display a distinct neurobiology in terms of brain structure and function [13].

PMDD is currently classified as a depressive disorder in the Diagnostic and Statistical Manual of Mental Disorders, 5th edition (DSM–5) [3]. Clinically, a clear distinction between depression and PMDD is often difficult to make, as both share a group of symptoms including depressed mood, hopelessness, lack of energy, sleep disturbance and diminished concentration [14, 15]. Despite the fact that a minimum of two months of prospective daily charting is required for an accurate diagnosis of PMDD [3], Craner and colleagues [16] reported that only 11.5% of physicians use 2-months of symptom reporting to diagnose PMDD, and only 18.4% regularly use any type of daily symptom rating. These findings indicate that it is critical to develop an assessment tool that is proven to be useful for clinicians in distinguishing between clinically significant PMS/PMDD and mood disorders, given the cyclic, temporal emergence of symptoms associated with PMDD. In spite of the high comorbidity between PMDD and mood disorders, we are unaware of any evidence-based tool that assesses both conditions concurrently.

In light of the aforementioned clinical and research gap in the assessment of PMDD in women with mood disorders, the objective of the current study was to develop a tool that prospectively monitors concurrent premenstrual and mood symptoms in women with mood disorders. Due to the rising awareness of women who suffer from both mood disorders and clinically significant PMS/PMDD, we have developed the “McMaster Premenstrual and Mood Symptom Scale (MAC-PMSS)” which is intended to better reflect the recently published DSM-5 criteria for monitoring both severe PMS/PMDD and mood symptoms.

## Methods

### Participants

Fifty-two females ages 16–45 were enrolled in the study. Participants were included in the study under the following criteria: (1) Diagnosis of BD or MDD according to the Structured Clinical Interview for DSM-IV-TR Axis I Disorders, Research Version, Patient Edition (SCID-I) [17]; (2) Presence of regular menstrual cycles (25–32 days). Participants were deemed ineligible from the study if they fulfilled any of the following exclusion criteria: (1) Irregular menstrual cycles; (2) Current or recent (last 6 months) alcohol or substance use disorder; (3) Current unstable general medical conditions.

Participants were recruited from the Women’s Health Concerns Clinic and the Mood Disorders Program at St. Joseph’s Healthcare Hamilton, Ontario, and from community advertisements in the greater Hamilton area. Community advertisements invited females aged 16–45, with a diagnosis of depression or bipolar disorder and regular menstrual cycles to contact our research staff if they were interested in participating in a research study. The study protocol was approved by the Hamilton Integration Research Ethics Board. All participants provided written informed consent in accordance with the Declaration of Helsinki prior to taking part in the study. Note that individuals aged 16 years or older could consent for research without parents’ assent.

### Design

All participants completed two study visits. At visit one (baseline), participants completed a standardized demographic and reproductive history, the SCID-I [17], and the SCID-PMDD [18]. Participants were then given two questionnaires to fill out at home: the MAC-PMSS and the Daily Record of Severity of Problems (DRSP) [19], a widely used clinical questionnaire to determine PMDD, including use in clinical trials [20–22]. Participants were asked to fill out the MAC-PMSS and the DRSP on a daily basis for two consecutive months, in order to capture two complete menstrual cycles. Participants were instructed to start the two months of prospective charting on the day after the baseline visit and, thus, they began the study at any phase of the menstrual cycle. After completing a minimum of two months of prospective symptom daily charting, participants came in for a second and final visit, where they returned the MAC-PMSS and DRSP logs and answered the following clinician-rated questionnaires: the Montgomery-Åsberg Depression Rating Scale (MADRS) [23], the Hamilton Depression Rating Scale [24], and the Young Mania Rating Scale [25], the most widely used questionnaires to measure severity of depressive and manic symptoms. They also filled out the premenstrual screening tool (PSST) [14].

### The MAC-PMSS

The MAC-PMSS was initially piloted in patients referred to the Women’s Health Concerns Clinic, St. Joseph’s Healthcare Hamilton and was also distributed to four clinician-scientists with expertise in women’s mental health for pilot use and feedback. Feedback from patients and experts was used to refine the questionnaire until the final version. The MAC-PMSS consists of two charts that are to be filled out simultaneously:

1. Mood symptom chart: A daily rating system enabling patients to track the severity of manic and depressive symptoms This mood chart was adapted from the National Institute of Mental Health-Life Chart Method (NIMH-LCM) [26]. Participants are asked to mark an “X” alongside the level of severity corresponding to their daily mood symptoms. Daily mood symptoms are subcategorized into “Stable, I feel fine” or four levels of severity of manic and depressive symptoms (“mild, moderate-low, moderate-high, severe”). Each category contains clinical descriptors that help participants choosing the most accurate symptom severity (e.g. “no functional impairment”, “unable to focus”, “pervasive low mood”, etc.). If participants experience both depressive and manic symptoms on the same day (mixed symptoms) they are oriented to mark both.
2. Premenstrual symptom chart: The premenstrual chart was adapted from the DRSP [19]. The wording was modified to be consistent with the wording from the DSM-5 criteria [3]. Participants are asked to record the levels of severity of symptoms according to a scale of 1–6 (1-not at all, 2-minimal, 3-mild, 4-moderate, 5-severe, 6-extreme), similar to the DRSP [19]. The first four symptoms correspond to the core symptoms included in the criterion “A” of the DSM-5 diagnosis of PMDD [3]. Participants were also asked to mark down days of menstrual bleeding or spotting, total hours they slept in the previous night and record any major life events that may have influenced the occurrence and/or the severity of their symptoms. Participants received monthly reminders to complete the MAC-PMSS, which was completed as a paper and pencil form.

The MAC-PMSS is copyright of McMaster University and is available at no cost to academic researchers and the general public, under the terms and conditions of a license agreement. A paid license is required for all Industry and commercial applications. Readers can access the questionnaire by emailing the McMaster Industry Liaison Office (MILO) at milo@mcmaster.ca or by completing the request form at https://research.mcmaster.ca/industry-and-investors/technologies-available-for-licensing/questionnaire-request-form/

### Statistical analyses

Pearson’s product-moment correlation was used to evaluate agreement of the premenstrual symptom severity between the individual items of the MAC-PMSS and the DRSP across two consecutive menstrual cycles.

In order to test the validity of MAC-PMSS in measuring severity of mood symptoms, we used Pearson correlations between depressive scores and MADRS/HAMD scores, and between mania scores and YMRS. The last seven days of mood ratings on the MAC-PMSS mood chart were used to correlate with MADRS, HAMD, YMRS because each of these mood questionnaires inquire about “the past week”.

The diagnosis of PMDD is confirmed when participants score ≥ 4 in at least five premenstrual symptoms, including at least one of the four main core symptoms, in the week before the onset of menses, starting to improve within a few days after the start of menses, which corresponds to scores ≤ 2 (which corresponds to a minimum of 50% improvement). Women who rate a level of extreme severity (score = 6) in their premenstrual symptoms would qualify for the diagnosis of PMDD if their symptoms are down to ≤ 3 (minimum 50% improvement). Note that the presence of subthreshold symptoms is common during periods of clinical remission in women with co-morbid MDD/BD [27, 28]. Two psychiatrists specialized in women’s mental health (BNF, LM) blinded to the participants’ diagnoses independently scored participants’ MAC-PMSS in order to confirm a diagnosis of PMDD or lack thereof. Interrater reliability was calculated using the extended percentage agreement among raters (package “irr” in R).

## Results

Fifty-two participants completed the study, of which 32 (61.5%) had a diagnosis of bipolar disorder and 20 (38.5%) had a diagnosis of MDD. An additional 40 participants were either a) deemed ineligible because they did not meet inclusion criteria or b) dropped out of the study due to a lack of compliance with log completion (did not complete two months of daily charting). The mean age of all participants was 32.4 ± 7.2 years. The average age at menarche was 12.2 ± 1.6 years. Overall, 31 (59.6%) participants met provisional diagnosis of PMDD according to the SCID-PMDD and 11 (21.1%) screened positive for PMDD according to the PSST. Five (9.6%) participants met PMDD criteria in 2 of 2 menstrual cycles and an additional 10 (19.2%) participants met criteria in 1 of 2 menstrual cycles prospectively according to the MAC-PMSS. Forty-four participants were on medications. Inclusive of all participants, the number of current psychotropic medications ranged from 0 to 6 (Table 1).

**Table 1:** Demographic characteristics

Demographic data (N = 52)	Mean (SD)
Age	32.4 (7.2)
Years of education	16.4 (2.4)
Diagnosis of major depressive disorder	20 (38%)
Diagnosis of bipolar disorder	32 (62%)
Age of onset of mood disorders	19.4 (6.7)
Number of psychotropic medications	1.92 (1.34)
Antidepressants, N	32 (62%)
Anxiolytics, N	10 (19%)
Antipsychotics, N	17 (33%)
Mood stabilizers, N	20 (38%)
Hypnotics, N	6 (12%)
Stimulants, N	3 (6%)
Age at menarche	12.2 (1.6)
BMI	26.8 (5.7)
	*N (%)*
Employed	36 (69.2)
Positive PMDD screen by PSST	11 (21.1)
Positive PMDD diagnosis by SCID-PMDD	31 (59.6)
Positive PMDD diagnosis by MAC-PMSS	5 (9.6%)
Current use of oral contraceptives	10 (19.2)
	Range
Total pregnancies	0–7
Number vaginal deliveries	0–2
Number C-sections	0–3
Number miscarriages	0–4
Number terminations	0–4

*PMDD* Premenstrual Dysphoric Disorder, *PSST* Premenstrual Symptom Screening Tool, *SCID-PMDD* Structured Clinical Interview for DSM-IV-TR, PMDD Assessment, *BMI* Body Mass Index

### Correlation between MAC-PMSS and DRSP scores

All of the individual items of the MAC-PMSS strongly correlated with the individual items of the DRSP across two consecutive menstrual cycles (p < 0.001; Table 2).

**Table 2:** Pearson’s *r* correlations and confidence intervals are provided for agreement between individual MAC-PMSS and DRSP items

Item	Late-luteal Cycle 1	Mid-follicular Cycle 1	Late-luteal Cycle 2	Mid-follicular Cycle 2
Depression	0.847 (0.739, 0.913)	0.640 (0.440, 0.779)	0.940 (0.896, 0.966)	0.979 (0.962, 0.989)
Anxiety	0.964 (0.935, 0.980)	0.955 (0.922, 0.974)	0.951 (0.913, 0.972)	0.964 (0.935, 0.980)
Mood swings	0.962 (0.932, 0.979)	0.815 (0.692, 0.891)	0.954 (0.919, 0.974)	0.944 (0.899, 0.970)
Anger/irritability	0.860 (0.758, 0.921)	0.952 (0.921, 0.974)	0.981 (0.966, 0.989)	0.941 (0.894, 0.968)
Loss of interest	0.859 (0.755, 0.921)	0.960 (0.930, 0.977)	0.966 (0.939, 0.980)	0.888 (0.801, 0.938)
Concentration	0.915 (0.851, 0.953)	0.955 (0.921, 0.974)	0.861 (0.765, 0.919)	0.957 (0.922, 0.976)
Energy	0.796 (0.659, 0.882)	0.813 (0.691, 0.890)	0.814 (0.691, 0.891)	0.815 (0.684, 0.895)
Appetite	0.823 (0.698, 0.899)	0.789 (0.653, 0.876)	0.896 (0.822, 0.941)	0.833 (0.711, 0.906)
Sleep	0.893 (0.813, 0.940)	0.462 (0.208, 0.658)	0.819 (0.698, 0.895)	0.740 (0.566, 0.851)
Overwhelmed	0.924 (0.865, 0.957)	0.954 (0.919, 0.974)	0.936 (0.888, 0.963)	0.982 (0.968, 0.990)
Physical	0.850 (0.741, 0.915)	0.906 (0.838, 0.946)	0.941 (0.897, 0.966)	0.777 (0.614, 0.870)

All results significant at p < 0.001

### Correlation between MAC-PMSS and depression/mania scores

Both the MADRS and the HAMD total scores significantly correlated with the MAC-PMSS depression scores (p < 0.01). Similarly, YMRS total scores significantly correlated with the MAC-PMSS mania scores (p < 0.01; Table 3).

**Table 3:** Pearson’s *r* correlations between MAC-PMSS and MADRS, HAMD, YMRS

	MAC-PMSS depression	MAC-PMSS Mania
MADRS	0.572	–
HAMD	0.555	–
YMRS	–	0.456

All results are significant at *p* < 0.01

*MADRS* Montgomery-Asberg Depression Rating Scale, *HAMD* Hamilton Depression Rating Scale, *YMRS* Young Mania Rating Scale

### MAC-PMSS agreement between independent rates

There was a high agreement (92.3%) between the two blinded independent raters who used the MAC-PMSS to confirm the diagnosis of PMDD.

## Discussion

Currently, there are no clinical tools that have been validated to prospectively monitor concurrent manic, depressive and premenstrual symptoms. In a well-characterized sample of women with mood disorders, we found that the MAC-PMSS has a high agreement rate compared to the most commonly used questionnaires for PMDD diagnosis (DRSP) and severity of manic (YMRS) and depressive symptoms (MADRS, HAMD). Coupled with high agreement rates between clinician-scientists specialized in women’s mental health, these results suggest that the MAC-PMSS is a useful tool to be used in clinical and research settings. The most valuable feature of the MAC-PMSS is the ability to simultaneously monitor the severity of manic, depressive and premenstrual symptoms on a daily basis throughout the menstrual cycle. Distinguishing between MDD/BD symptoms/episodes and clinically significant premenstrual symptom worsening is crucial when assessing diagnosis and choosing the most appropriate treatment plan [29, 30]. In the clinical practice, it is not uncommon that we hear the frustration of women with co-morbid mood disorder and PMDD that their premenstrual worsening has been overlooked by their health care providers, who tend to focus on the mood disorder treatment and fail to identify and subsequently offer effective treatments for premenstrual symptoms [16, 31]. Similarly, it is also not uncommon to assess women referred for “PMDD” who end up being diagnosed with a different disorder (e.g. MDD, BD, borderline personality disorder) after proper two-month prospective daily charting. This is consistent with studies showing low agreement rates between retrospective- and prospective-based clinical assessments [32], which supports the DSM-5 recommendation of two-month prospective daily symptom charting for an accurate diagnosis of PMDD. Note that while many studies used diaries designed specifically to assess premenstrual symptoms, there was limited overlap between the diaries used which affects the comparability of studies [33, 34].

When assessing possible diagnosis of PMDD, it is also important to keep in mind that even healthy young females have significant variability in their mood patterns across their menstrual cycles [35]. However, women tend to report on premenstrual symptoms only when they are asked [36]. Although the monthly episodes of PMDD tend to be short-lived, these PMDD symptoms cause a significant negative impact on well-being and quality of life [37]. They should not be considered simply as an exaggeration of mood patterns occurring in healthy women. It has been suggested that addressing deviations in mood patterns from individual women’s own affective norm would have greater clinical utility than comparing their symptoms with others [35]. Thus, a careful diagnosis including prospective daily charting is critical for proper assessment and management of individuals with comorbid PMDD and mood disorders so they can regain full functioning. In our study, 9.6% of the study participants met prospective criteria for PMDD in two consecutive menstrual cycles, while an additional 19.2% met PMDD criteria in 1 of 2 menstrual cycles, some of which would likely meet criteria in two symptomatic cycles should the daily symptoms tracking continued longer than two months. Our results are consistent with a large epidemiological study that reported a 22 ± 9% co-morbidity rate between mood disorders and PMDD [10].

A related area of research that requires attention is the differentiation between co-morbid PMDD versus premenstrual exacerbation of an underlying mood disorder. According to the International Society for Premenstrual Disorders (ISPMD), “premenstrual exacerbation occurs when there is magnification of an underlying somatic, medical or psychiatric disorder during the luteal phase of the ovarian cycle. The profile of symptoms is similar throughout the cycle but the intensity is significantly greater in the premenstrual phase” [38]. Previous reviews on this topic have highlighted the various methodological flaws of previous studies, which led to over- or underestimates of the prevalence of premenstrual exacerbation of mood disorders [39, 40]. Notwithstanding these methodological limitations, available evidence suggests that premenstrual exacerbation may occur in 44–68% of individuals with mood disorders [39].

From a clinical standpoint, this study attempts to fill an important gap, which is the disconnect between research and clinical practice. While research studies are very clear that PMDD diagnoses based on retrospective reports yield high rates of false positives [41], which supports the need of prospective daily charting for an accurate diagnosis of PMDD, surveys from patients [42] and health care providers [16] show a staggering different reality in clinical practice. Specifically, a recent survey of over 2500 patients who sought help from general practitioners, psychiatrists, gynecologists or psychotherapists for PMDD symptoms reported that in 68% of cases the diagnosis of PMDD was *not* confirmed through prospective charting [42]. Similarly, a study that surveyed 87 outpatient physicians found that only 11.5% routinely collected symptom ratings for 60 days to confirm the diagnosis of PMDD [16]. Part of the disconnect between the requirement of 60-day daily symptom charting for an accurate assessment of the diagnosis of PMDD and the clinical challenges implementing this recommendation in the real-world clinical practice is that the algorithms used to diagnose PMDD in research are not user-friendly for routine use in the clinical practice [43]. Therefore, because since the start of this project we envisioned the development of a user-friendly tool that could be widely implemented in the clinical practice, the MAC-PMSS attempts to bridge this gap between research and the clinical practice. This goal was possible through the feedback from real patients from a well-established women’s mental health clinic and from clinician-scientists with extensive expertise in this area. We firmly believe that the involvement of people with lived experience early in the development of this clinical tool is a major strength. The MAC-PMSS has been used since its copyright in 2014 as the official clinical tool to prospectively track premenstrual and mood symptoms in a major health care center in southern Ontario (https://www.stjoes.ca/hospital-services/mental-health-addiction-services/mental-health-services/women's-health-concerns-clinic-whcc). Current efforts are underway to turn the paper and pencil version of the MAC-PMSS tool into an app, which will further improve access and acceptability of this new clinical tool. The development of automated systems for collection of daily ratings with standardized scoring and reporting of results to clinicians has been identified as a major step forward in this field [43]. However, despite its several years of clinical use as a diagnostic tool for PMDD in patients referred to a specialized mental health clinic, the MAC-PMSS has only been formally validated in individuals with BD and MDD. Thus, future studies should test and validate how this new clinical tool would perform in non-mood disorder populations.

Some limitations to the current study are noteworthy. First, like other self-rating diaries, there is a possibility of inaccurate reporting or that the responses may be confounded by the cycling nature of their depressive or bipolar disorders. However, the comparison with the DRSP and other mood severity rating scales increases the confidence of our results. Second, although our sample size was modest, our sample size is comparable to many other previous studies used to validate premenstrual symptom scales [44–49]. Another limitation is that our study is consistent with many previous studies describing the challenges that participants face filling out daily symptom charting for two months [50–52]. Many participants were excluded from the study because they ceased charting prematurely for various reasons including failure to recall completing the chart on any given day (or a period days), unintentionally rendering the charts unusable (e.g., coffee spill on paper, crumpling, etc.), or other commitments/priorities that took precedence. Notably, we have leveraged this feedback in the design of a mobile application to facilitate enhanced support for completion of charting in a more acceptable format.

## Conclusion

The MAC-PMSS has a high agreement rate compared to the most widely used questionnaires to measure premenstrual symptoms, and severity of manic and depressive symptoms, which indicates that the MAC-PMSS is a reliable tool to prospectively monitor concurrent mood and premenstrual symptoms.

## Data Availability

The datasets generated and analysed during the current study are not publicly available due to the fact that the research participants did not consent for their data to be shared with other research groups, but are available from the corresponding author on reasonable request.

## Abbreviations

BD: Bipolar disorder
DRSP: Daily Record of Severity of Problems
DSM-5: Diagnostic and Statistical Manual of Mental Disorders, 5th edition
HAMD: Hamilton Depression Rating Scale
MAC-PMSS: McMaster Premenstrual and Mood Symptom Scale
MADRS: Montgomery-Åsberg Depression Rating Scale
MDD: Major depressive disorder
NIMH-LCM: National Institute of Mental Health-Life Chart Method
PMDD: Premenstrual dysphoric disorder
PMS: Premenstrual syndrome
PSST: Premenstrual screening tool
YMRS: Young Mania Rating Scale
